# Aligning entity of knowledge graph based on graph convolutional network with maximum translated similarity

**DOI:** 10.1371/journal.pone.0362390

**Published:** 2026-11-06

**Authors:** Jianhui Zhou, Yun Gao, Xiangbing Zhao, Yanping Guo, Xulong Zhang

**Affiliations:** School of Computer and Network Engineering, Shanxi Datong University, Datong, China; Instituto Nacional de Pesquisas Espaciais, BRAZIL

## Abstract

Entity alignment is a pivotal technology for integrating heterogeneous knowledge graphs, yet most embedding-based alignment methods destroy the inherent structural embedding information of knowledge graphs when unifying cross-graph embedding spaces, resulting in limited alignment accuracy and stability. To tackle this issue, this paper proposes a novel model for entity alignment tasks, which named as Graph Convolutional Network with Maximum Translated Similarity (GCN-MTS). GCN-MTS innovatively leverages a maximum translated similarity mechanism to optimize the adjacency matrix, degree matrix and loss function. This core design effectively maintains the original relative embedding structure of cross-graph entities during embedding space unification, which is the key improvement over existing approaches. Comprehensive experiments on 3 pairs of real-world knowledge graph dataset demonstrate the superior performance of GCN-MTS against advanced baseline methods. It achieves average performance gains of 5%–15% in Hits@1 and 4%–16% in Hits@10. Ablation results confirm that the optimized module brings a nearly 22% improvement in alignment metrics compared with the vanilla GCN. Furthermore, GCN-MTS presents excellent robustness and stability across different seed alignment quantities and seed combinations.

## 1. Introduction

Knowledge graph (KG) is effective knowledge representation model that have been widely applied various scenarios, including recommender systems, question answering systems, text generation systems, and knowledge reasoning systems [1,2]. Numerous KGs have been constructed as knowledge base for the aforementioned applications. Merging multiple KGs can produce a large-scale unified KG, which provides richer knowledge representation model for special applications [3,4]. In different KGs, same entities existed in different labels such as *lgd*:*240111203* in source KG and *dbp*:*Kromsdorf* in target KG, as shown in Fig 1. It is an important work for merging KGs that recognizing same real-world entities accurately in them [5].

**Fig 1:**
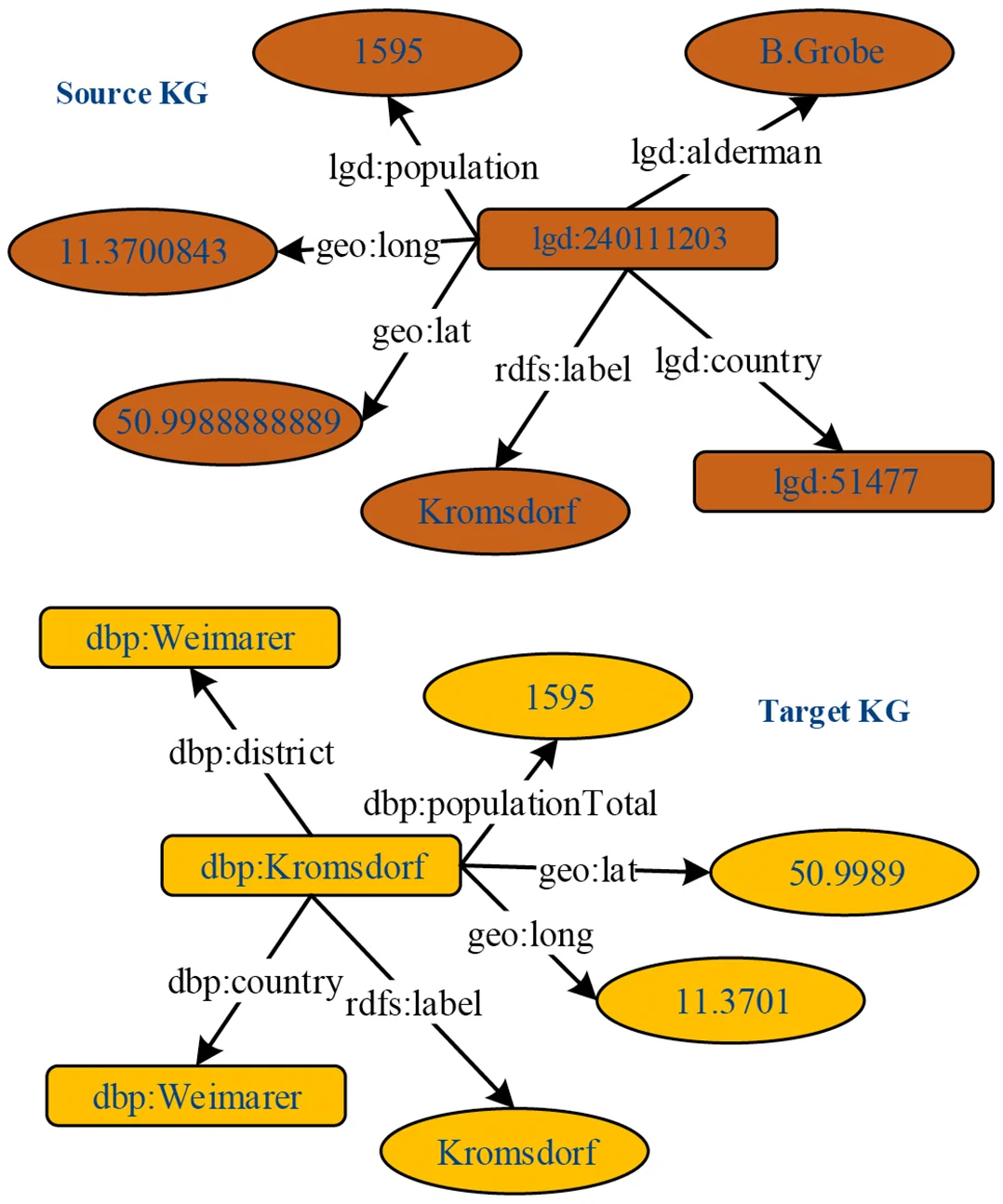
Instance of little piece of KGs.

Entity alignment is a crucial research topic, whose essence is recognizing same entities across heterogeneous KGs [6–8]. A KG is typically represented as a set of 3-tuples, for instance, (*dbp*:*Kromsdorf*, *dbp*:*country*, *dbp*:*Weimarer*) is a 3-tuple in target KG, as shown in Fig 1. In a 3-tuple, a head entity was correlated to a tail entity with a relationship, for instance, *dbp*:*Kromsdorf* and *dbp*:*Weimarer* are the head entity and the tail entity respectively, and *dbp*:*country* is the relationship between them [9]. In this paper, we studied the technology of entity alignment, and used 3-tuple form to recognize same real-world entities across different KGs.

Early research on entity alignment mainly focused on calculating the similarity of entity pairs, including string similarity and structure similarity [10–12]. These methods rely heavily on manually designed rules [13]. Such rule-based approaches are not only labor-intensive but also suffer from limited performance, due to the inherent irregularity and heterogeneity of real-world KGs [14]. For example, *lgd*:*240111203* and *dbp*:*Kromsdorf* are same real-world entities. But, string similarity between them is very low. Because of *dbp*:*Kromsdorf* has a relationship *dbp*:*district* but *lgd:240111203* does not have, structure similarity between them is not high.

In recent years, entity embedding techniques have been widely used for KG entity alignment, including typical models such as TransE, TransH, and TransR [15,16]. Graph Convolutional Network (GCN) are also utilized to learn structural features of KGs and generate entity embeddings [17]. Semantic similarity could be calculated by distance between embedding vectors of entities [18]. Although these methods enhanced performances of entity alignment, the key factor of them is how to make entity embeddings to fall in same vector space [19,20]. To address this issue, seed alignments were adopted to resolve this problem. Entities were embedded by machine learning algorithm at the constraint condition of determined seed entities [21–23]. In the algorithms, common objective function was formulated for source KG and target KG to make sure that entity embeddings in them falling in same vector space [24–26]. However, it was destroyed that the relative position between entities in source KG or in target KG. During embedding entities in same vector space, the embeddings of some entities are re-determined under the constraint of seed anchor entities, that leads to drastic shifts in their relative positional relationships within the new space, which further breaks the original semantic correlations between paired entities. For instance, considering target KG only, the distance between *dbp*:*Kromsdorf* and *dbp*:*Weimarer* was 5 by TransE. If *dbp*:*Kromsdorf* was re-determined by seed entities, the distance between them might not be 5 anymore.

In this paper, Maximum Translated Similarity (MTS) model is presented to enhance GCN model, whose name is Graph Convolutional Network with Maximum Translated Similarity (GCN-MTS). GCN-MTS make entity embeddings falling in same vector space at the condition of keeping the relative position maximumly. Moreover, for that reason, performance of the proposed method was more stable, although using different seed alignment or different numbers of seed alignment. The main contributions made in this paper are summarized as follows:

- The novel model, i.e., MTS is proposed and applied.
- The framework of the method of aligning entity based on GCN-MTS is proposed.
- The proposed method is evaluated by 3 pairs of KGs. The results show that the proposed method has higher performance than the state-of-art methods.

The rest of the paper is organized as follows: Section 2 describes the related works; Section 3 specifies the method which contains MTS and GCN-MTS, and elaborates the process of aligning entity based on GCN-MTS; Section 4 shows the experimental results, and evaluates the proposed method comparing with the state-of-art methods; finally, Section 5 and section 6 draw the discussion and conclusion.

## 2. Related works

As mentioned in previous section, approach of entity alignment mainly refers to rule-based method and embedding-based method. In this section, the research review of them is summarized as follows.

### 2.1. Rule-based entity alignment

Rule-based entity alignment approaches mainly adopted string similarity and structure similarity between entities. Some methods or models of calculating similarity were proposed based on artificial rules.

MOMIS [27] specified mapping rules and integrity constraints to integrate heterogeneous information sources, and the mapping rules were described by ODL schema. COMA [28] combined Affix, n-gram, EditDistance, and Synonym to detect duplicate database records concerning the same real-word entity. SAMBO [29] implemented several matchers based on linguistic matching, structure-based strategies and constraint-based approaches to align entities in ontology. LIMES [30] presented the mathematical foundation named Distance Approximation based on the Triangle Inequality for discovering large-scale links on the Web of Data. OARS [31] demonstrated a combinational approach to handle uncertain entities in ontology alignment, which considered both lexical and structural similarity measures. CLEM [32] employed Jaccard similarity with character-level n-grams to match cross-lingual entities and align infobox in Wikipedia, that compare the string portion of attribute values with token sets. LinkWeight [33] used semantic characteristics of two entities and Google page count to calculate an information distance similarity between them. Terminological Similarity, Structural Internal Similarity, and Structural External Similarity were employed to compute the overall similarity. An automatic string alignment algorithm was presented in [34] to detect links between the TheSoz and DBpedia, it relied on the binary string match with some normalization preprocessing. SIMTSS [35] found the correspondence links between entities in two intrinsically heterogeneous ontologies, which adopted different methods of calculating string and structure similarities.

As mentioned above, the existed rule-based approaches mainly calculated string similarity and/or structure similarity of entities. String similarity generally requires uniform criteria of entity label, and using some external resources. Structure similarity generally requires isomorphic KGs. But the real situation is that KGs had been built for diverse purposes, and so regularity and homology between KGs are not assured.

### 2.2. Embedding-based entity alignment

Embedding-based entity alignment approaches mainly generated embeddings of each entity, and then calculated embedding distance to identify same entities between KGs [36]. Embeddings preserve the information of semantic relationships between entities in KG, which can be used for entity alignment. Graph Neural Networks and Graph Convolutional Network have become the dominant methods for network alignment, where anchor or seed links are widely adopted as supervised guidance to make the embedding space consistent between source and target networks.

MCEA [37] presented a multiscale convolutional graph network, which implemented a semi-supervised learning by the proposed graph embedding model. A larger graph convolution kernel was used to preserve the information of multi-hop neighbor entities in the model. DAGCN [38] pre-trained preliminary embeddings of entities using seed entity embedding initializer. Heterogeneity entropy of degree was defined to measure the degree difference of entities, that was joint with alignment to make adversarial training for entity alignment. EAMI [39] was an entity alignment model based on multiple information, whose included string, structural and semantic information. Structural embedding and attribute embedding were learned by highway graph convolutional networks, and then they were joint to generate embedding similarity matrix. FuAlign [40] firstly fused source KG and target KG based on the seed alignments, and then learned semantic embeddings, long-term embeddings, context embeddings, finally obtained one-to-one alignments by a reliability-based stable matching algorithm. GAEA [41] developed a method which exploited attribute and structure information to embed entities in KGs. Noise data in KG can be fitted by this method. GAEA utilized limited entity pairs with noisy samples to deal with entity alignment. SDHN [42] built a Teacher-Student Network and a Rotation and Penalty module. Teacher network fused semantic information into student network to improve entity embeddings. It reduced the impact of noise data. Rotation and Penalty module enhanced the performance of entity alignment. EASA [43] utilized entity embeddings for semantic aggregation and attribute attention. It generated semantic aggregation of entities by different attributes, and distinguished different roles of attributes by attribute attention. EMGCN [44] was a graph convolutional network, who learned relation-aware embeddings by extracted relationships from KG. It consisted of several layers. Each layer encoded networks at multiple orders. On this basis, EMGCN deal with entity alignment by relational alignment and attribute alignment. Self-MEA [45] aligned entity by a self-supervised form. It was a method of multi-modal contrastive learning, which using relations embedding and attribute embedding. Self-MEA provided an insight of avoiding expensive labelling cost and breaking the information unicity of single-modal KG. SVNM-GAT [46] consisted of subgraph discriminator, multi-content graph attention network and virtual matching mechanism. Weighted adjacent matrix and entity embeddings were obtained by subgraph discriminator and multi-content graph attention network, respectively. Taking them as input and implementing virtual matching mechanism, the long-range dependency of entities was captured considerably. To address structural heterogeneity between networks, Salign [47] transfers high-order graphlet features across networks via anchor node pairs, which designing a cross-network attention module to mitigate over-smoothing defects of standard GNNs under sparse anchor supervision. BRIGHT [48] constructs a unified embedding space by random walk with restart centered on anchor nodes, which theoretically analyzing the space disparity problem of embedding-based alignment models and proposing a flexible anchor-based optimization objective for both plain and attributed networks. Clean-Anchor Alignment [49] is a filtering module integrated into GNN encoders to eliminate noisy and mismatched anchor pairs, which significantly improves the stability of alignment results on noisy social media and biological PPI datasets. APLEA [50] introduces anchor-aware position encoding based on random walk with restart, excavating implicit structural correlations between anchor nodes and common entities to compensate for limited labeled anchor signals in knowledge graph alignment tasks. TALP [51] is a two-layer graph attention network for heterogeneous cross-network anchor link prediction, that discard local type features. TALP jointly captures local type-aware signals and global topological information under one unified optimization objective, which effectively improves anchor matching performance on heterogeneous social networks.

As mentioned above, the existed embedding-based approaches mainly used embeddings of entities to implement supervised learning. Because the vector space of entity embeddings in source KG is different from that in target KG, the approaches mentioned above developed several fused or jointed methods to make embeddings falling in same vector space. But the relationship embeddings of source KG or target KG were destroyed by these approaches. For the purpose of enhancing performance of entity alignment, this paper proposes the GCN-MTS model to make KGs falling in same vector by the constrain of preserving their relationship embeddings.

## 3. Methodology

### 3.1. Overview of embedding entities with GCN-MTS

To align entities of source KG and target KG, seed entities, TransE, GCN, and GCN-MTS were deployed in this paper for embedding entities into the same space. As shown in Fig 2, the proposed method includes 4 steps.

**Fig 2:**
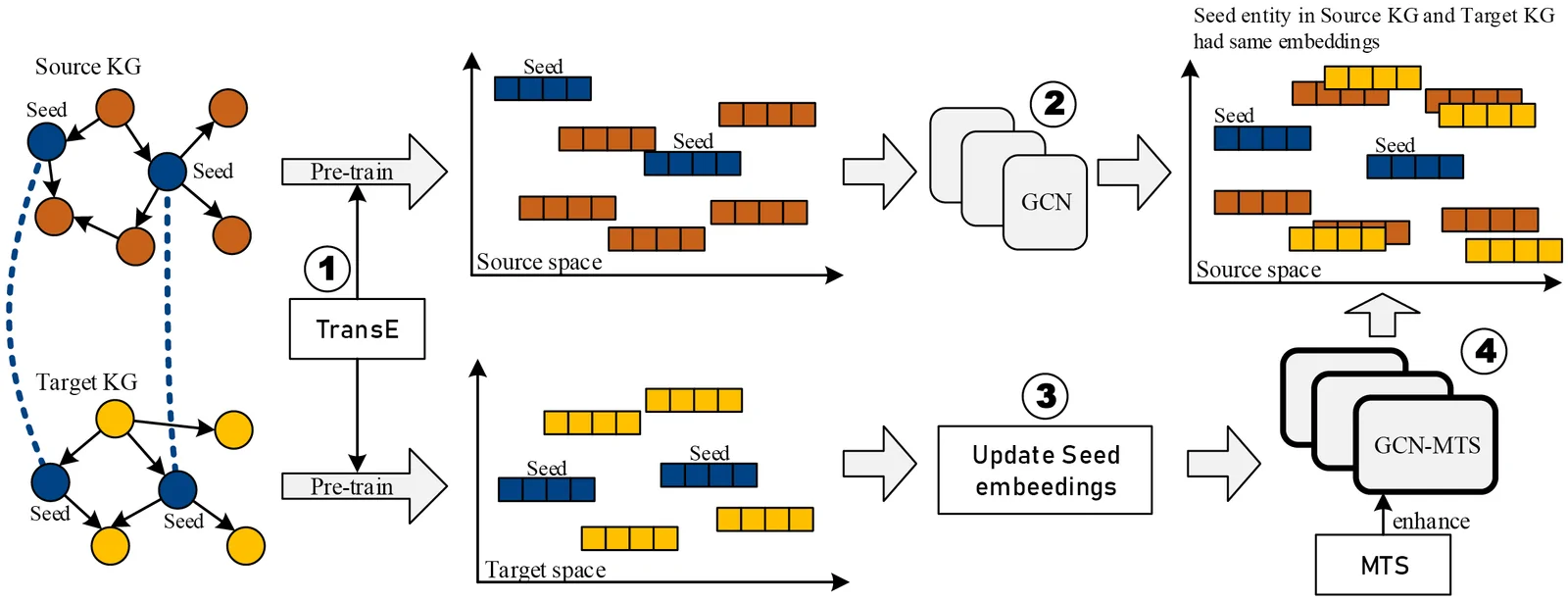
The framework of embedding entities with GCN-MTS.

The inputs of the method contained source KG, target KG, and seed entities. For more convenient elaboration in this paper, seed entity was named as Seed; entity of KG besides Seed was named as Translated Entity (TRE); TRE of source KG was named as Source Entity (SE); TRE of target KG was named as Target Entity (TE).

**Step 1. Pre-training by TransE**

In this step, source KG and target KG were pre-trained by TransE, respectively. Therefore, the embedding of Seed in source KG was not equal to the embedding of correspondent Seed in target KG probably. In other words, as shown in Fig 2, Seed of source KG fell into source space, and Seed of target KG fell into target space after this step.

**Step 2. Enhancing the embeddings of SEs by GCN**

In this step, GCN was deployed for enhancing the embeddings of SEs. The structure information of source KG was learned richly, as shown in Fig 2, Seeds were denoted as blue color, and SEs were denoted as orange color.

**Step 3. Updating the embeddings of Seeds in target KG**

In this step, the embeddings of Seeds in target KG were updated to equal the embeddings of Seeds in source KG. As shown in Fig 2, the Seeds in target KG and which in source KG had the same embeddings in source space.

**Step 4. Enhancing the embeddings of TEs by GCN-MTS**

In this step, GCN-MTS was deployed for enhancing the embeddings of TEs. The structure information of target KG was learned richly, and the relationships between entities in target KG were furthest preserved. In other words, as shown in Fig 2, TEs were denoted as yellow color, and the relative location among them in target space were furthest preserved after they were embedded into source space by GCN-MTS. In GCN-MTS, the MTS was combined. The specification of MTS and GCN-MTS were illustrated in 3.2 section and 3.3 section, respectively.

### 3.2. Specification of MTS

A KG is composed of a set of 3-tuples, which are denoted as Equation 1. Where *h*_*i*_ is head entity of 3-tuple; *t*_*i*_ is tail entity of 3-tuple; and *r*_*i*_ is relationship between *h*_*i*_ and *t*_*i*_.


$$\begin{array}{c} \text{KG}=\{(h_{i},\;r_{i},{\;t}_{i})|i\;\in \;N\} \end{array}$$ (1)

Each entity in KG can be represented as a vector by the entity embedding algorithm, such as TransE, TransH, TransR, etc. The vector that connects two entities implies semantics of relationship between them. As shown in Fig 3, There is a 3-tuple (*h*, *r*, *t*). If the head entity *h* and the tail entity *t* are translated to *h*’ and *t*’ by the vector *a*_1_ and *a*_2_ respectively, the translated similarity of (*h*, *r*, *t*) and (*h*,’ *r*,’ *t*’) is equivalent to the similarity of *r* and *r*,’ which can be calculated by Equation 2.

**Fig 3:**
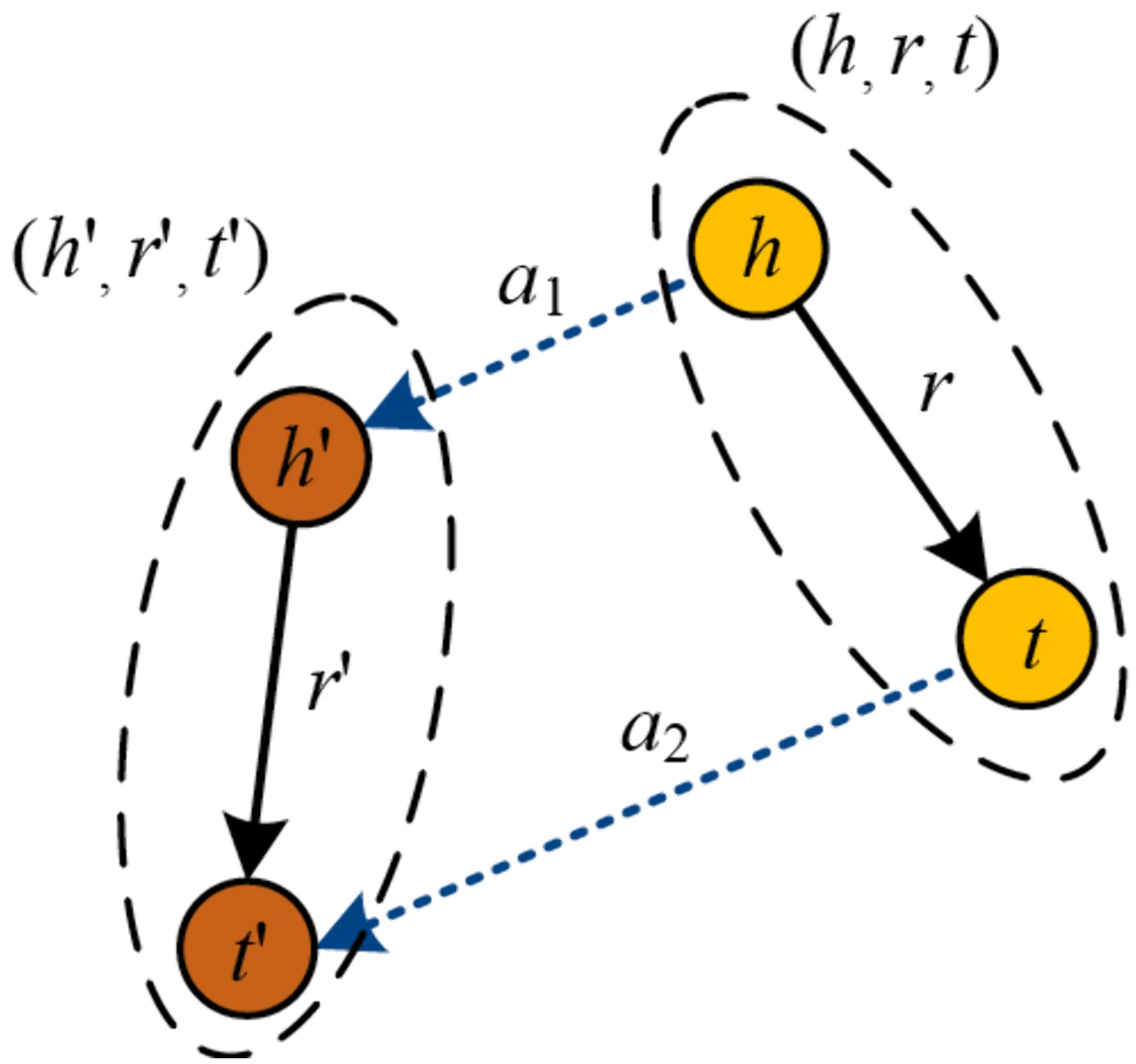
Translated similarity in simple situation.


$$\begin{array}{c} \text{sim}(r,\;r\prime )=\eta_{\text{1}}\text{cos}\theta +\frac{\eta_{\text{2}}}{{\left(\right|r\left|-\right|r\prime \left|\right)}^{\text{2}}} \end{array}$$ (2)

Where *θ* is the angle between *r* and *r*’; *η*_1_ and *η*_2_ are the hyperparameters.

Considering a general situation as in Fig 4, there is the KG who is composed of {(*h*_1_, *r*_1_, *t*_1_), (*h*_2_, *r*_2_, *t*_1_)}. If the entities *h*_1_, *h*_2_, *t*_1_ are translated to *h*_1_,’ *h*_2_,’ *t*_1_’ by different vectors *a*_1_, *a*_2_, *a*_3_ respectively, i.e., the KG is translated to KG,’ the translated similarity of KG and KG’ can be calculated by Equation 3.

**Fig 4:**
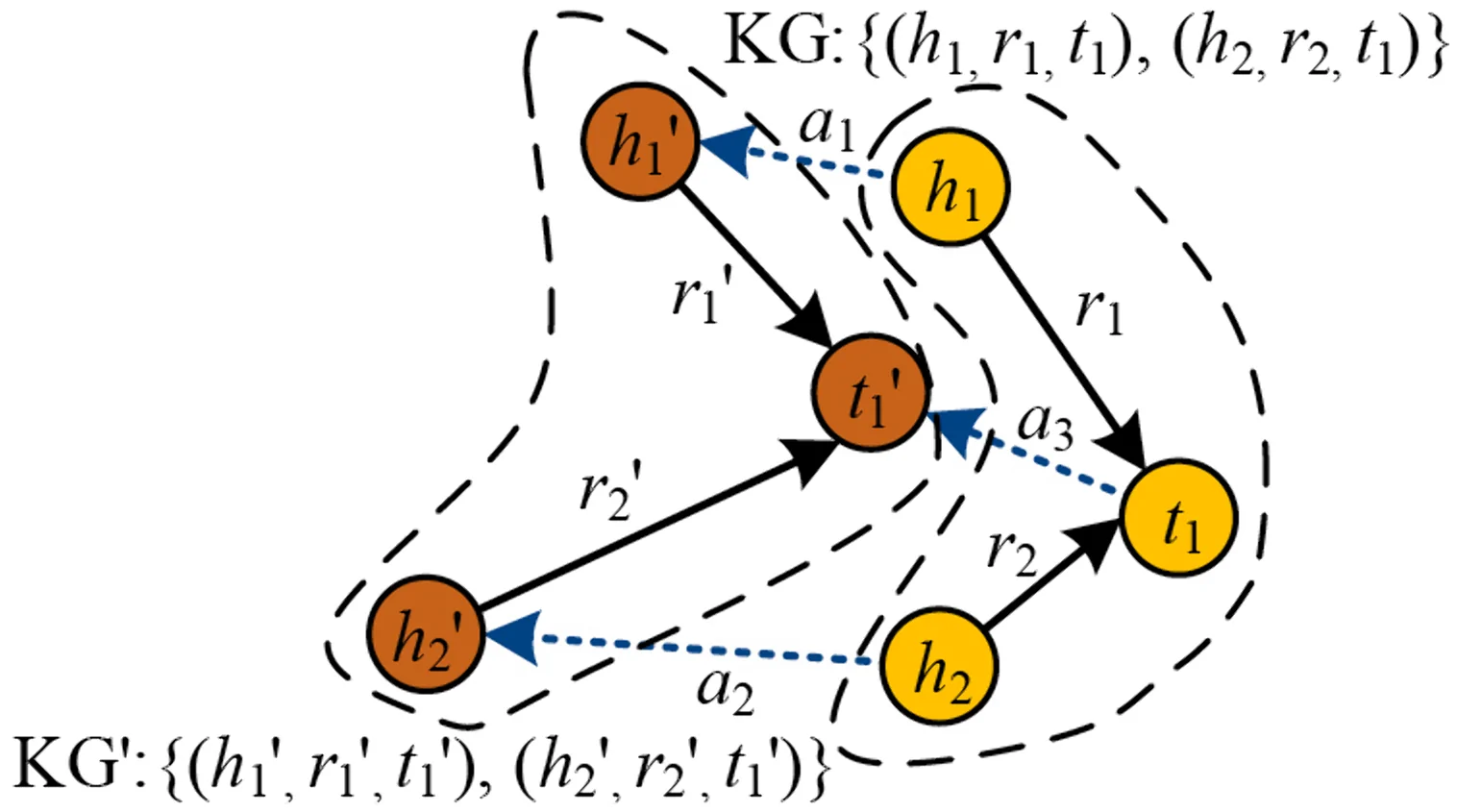
Translated similarity in general situation.


$$\begin{array}{c} {\text{s}\text{im}}_{\text{T}}(\text{KG},\text{ KG}\prime )=\frac{\text{1}}{\text{2}}[\lambda^{h_{\text{1}}}\text{sim}(r_{\text{1}},\;r_{\text{1}}\prime )+\lambda^{h_{\text{2}}}\text{sim}(r_{\text{2}},\;r_{\text{2}}\prime )] \end{array}$$ (3)

Where $\lambda^{h_{\text{1}}}$ is the importance coefficient that refer to the importance of *h*_1_ to *t*_1_; in the same way, $\lambda^{h_{\text{2}}}$ refers to the impor*t*ance of *h*_2_ to *t*_1_. How to calculate *λ* will be described in the section 3.3.

Generalizing Equation 3 to the KG composed of *n* 3-tuples, the translated similarity of KG and KG’ can be calculated by Equation 4.


$$\begin{array}{c} {\text{sim}}_{\text{T}}(\text{KG},\text{ KG}\prime )=\frac{\text{1}}{n}\sum_{i=\text{1}}^{n}\lambda^{h_{i}}\text{sim}(r_{i},\;r_{i}\prime ) \end{array}$$ (4)

Where *n* is the number of 3-tuples in KG; *r*_*i*_ is the relationship in the *i*^th^ 3-tuple; *r*_*i*_’ is the relationship that *r*_*i*_ translated; $\lambda^{h_{i}}$ is the importance coefficient of the head entity *h*_*i*_ to the tail entity *t*_*i*_ in the *i*^th^ 3-tuple.

To embed target KG into the source space by preserving the relationships itself, as shown in Fig 5, *e*_1_ and *e*_2_ have to be translated to *s*_1_ and *s*_2_, respectively. The other entities in target KG will be translated to the optimal coordinates, which make sure that the translated similarity of target KG and KG’ is as greater as possible.

**Fig 5:**
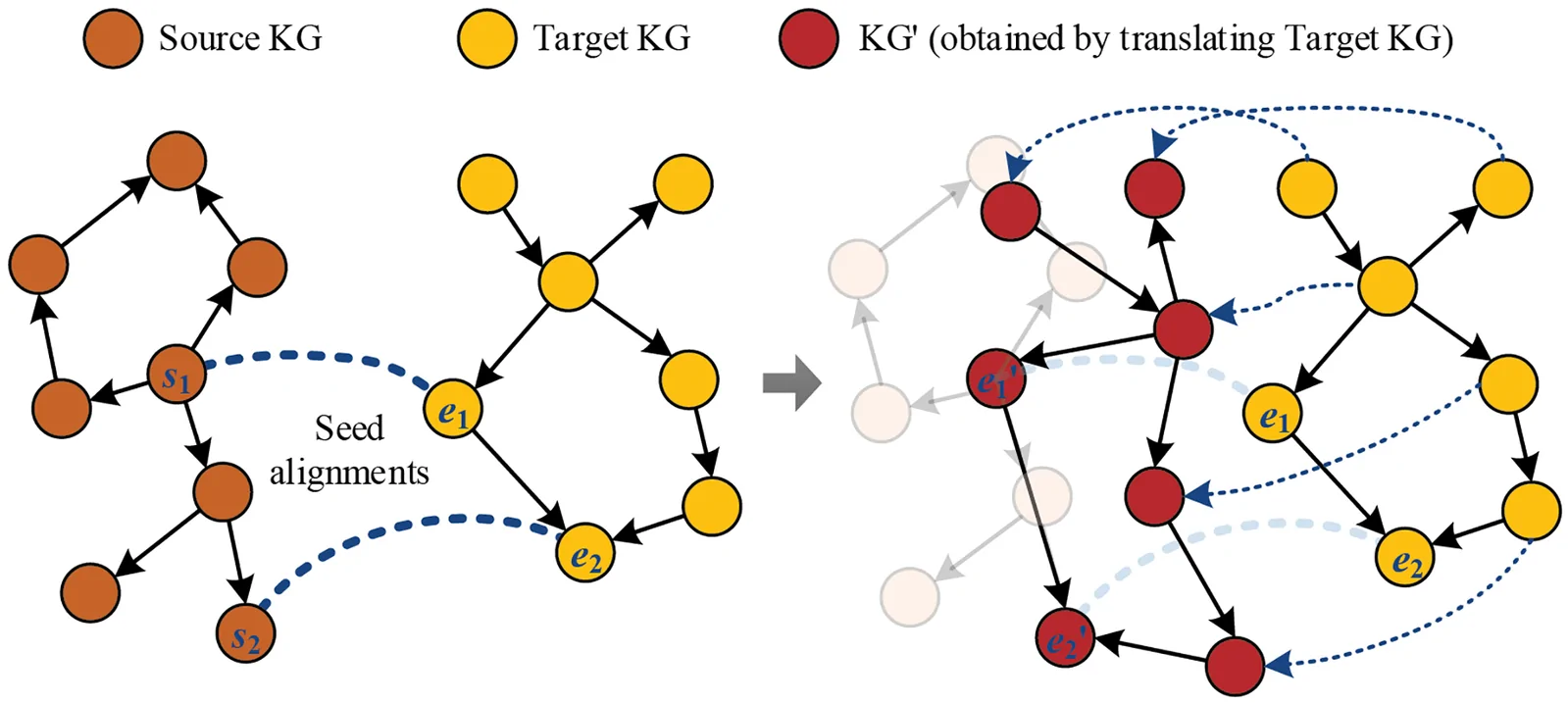
Diagram of translating entities based on MTS.

Therefore, MTS is denoted as follows:


$$\begin{array}{c} \text{MTS}:=\left\{ \begin{array}{c} @l\text{max}\text{imize}\;f(e_{i})\;=\;{\text{sim}}_{\text{T}}\left(\text{target KG},\text{ KG}\right)\\ \text{s}.\text{t}.\;e_{j}\;={\;s}_{j}\;\;\; \end{array}\right. \end{array}$$ (5)

Where *e*_*i*_ are the entities in target KG besides the seed entities; *e*_*j*_ and *s*_*j*_ are the seed entities in target KG and source KG, respectively.

### 3.3. Specification of GCN-MTS

Entity embedding is fused the information of all other entities in KG through multiple iterative learning of GCN. To preserve partial structure of target KG after it is translated into source space, GCN-MTS partitioned target KG into several sub-KGs according to its Seeds. By calculating the distance between each TE and Seed, the category of TE was determined. TE was classified into the category of Seed distance between which is smallest. For instance, as shown in Fig 6, the distance between TE_1_ and Seed_1_ is smaller than which between TE_1_ and Seed_2_, so TE_1_ was classified into the category of Seed_1_. Target KG was partitioned into sub-KG_1_ and sub-KG_2_. Embedding of TE_1_ took more account of the information of other entities in sub-KG_1_. That means Seed_1_, TE_2_, and TE_3_ had more influence on TE_1_ than Seed_2_, TE_4_, and TE_5_.

**Fig 6:**
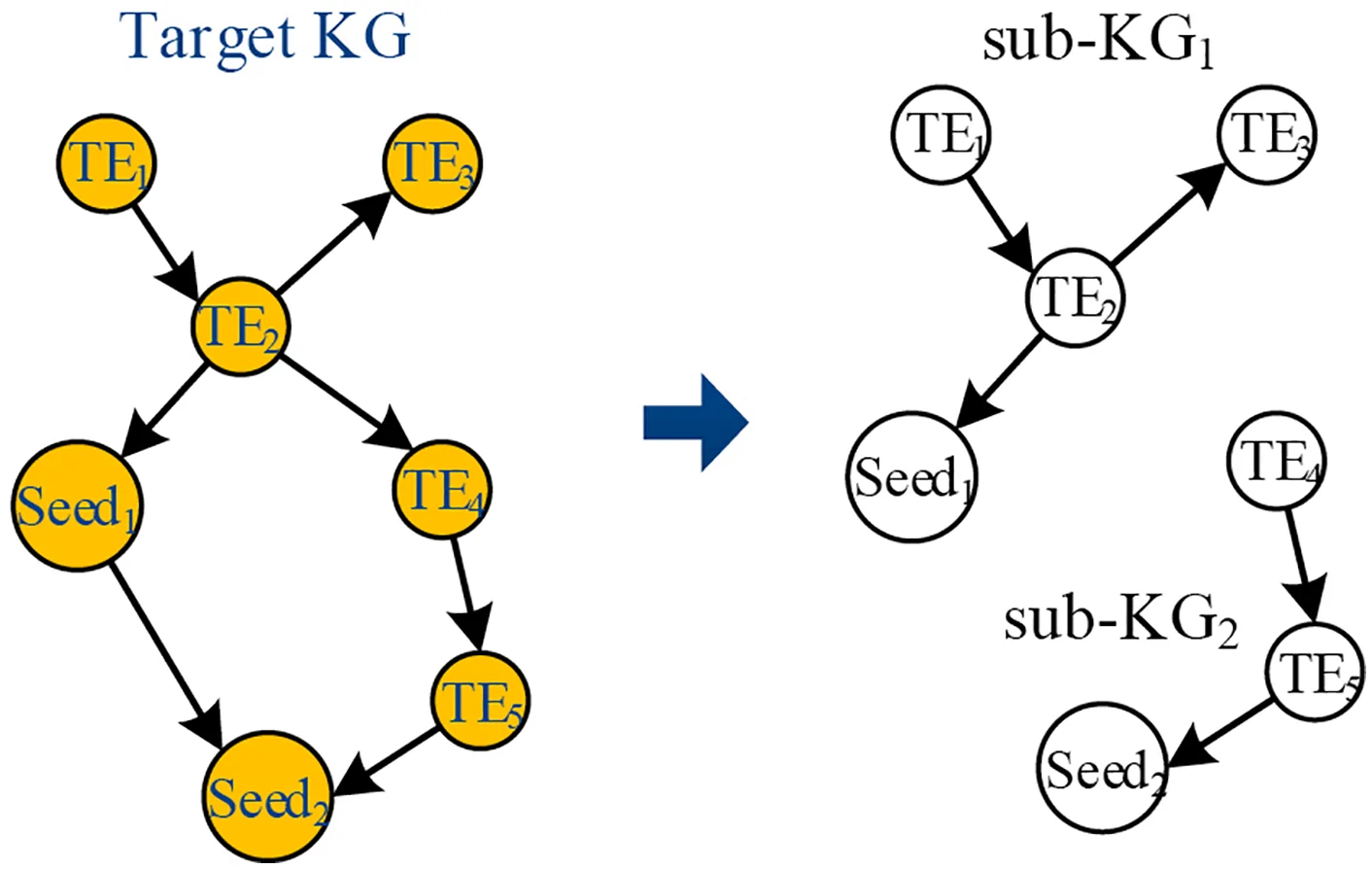
Diagram of partitioning target KG into sub-KGs.

**Adjacent matrix**. According to the results of partitioning target KG, the adjacent matrix **A** was redefined by all the sub-KGs as follows:


$$\begin{array}{c} 𝐀.\text{arcs}[i][j]=\left\{ \begin{array}{c} @l\text{1}\;\;\text{ if }i,\;j\text{ has a relationship and in same category}.\\ \text{0}\;\;\text{ else}.\;\;\;\;\; \end{array}\right. \end{array}$$ (6)

Importance coefficient reflects how important the translated entity is. The more important the TE is, the more influence it has on the translated similarity of target KG. In this paper, two factors were taken into account, which are numbers of relationships (NR) and distance between TE and Seed in same category (DS).

If a TE had more relationships, it was more important in target KG. The importance coefficient of NR of TE, i.e. *λ*_NR_(TE) was calculated by Equation 7. Where NR(TE) is the numbers of relationships the TE has.


$$\begin{array}{c} \lambda_{\text{N}\text{R}}\left(\text{TE})=\text{log}(\text{NR}(\text{TE})+\text{1}\right) \end{array}$$ (7)

In previous section, target KG was partitioned into several sub-KGs. Each sub-KG had only one Seed as its center. If a TE was closer to the center of its sub-KG, it was more important in target KG. The importance coefficient of DS of TE, i.e. *λ*_DS_(TE) was calculated by Equation 8. Where Dis(TE, Seed) is the distance between TE and Seed in same sub-KG, i.e., in same category.


$$\begin{array}{c} \lambda_{\text{D}\text{S}}(\text{TE})=\text{1}/e^{\text{Dis}(\text{TE},\text{ S}\text{eed})} \end{array}$$ (8)

Importance coefficient of TE was calculated by summing *λ*_NR_(TE) and *λ*_DS_(TE). In Equation 9, *λ*(TE) is the importance coefficient of TE.


$$\begin{array}{c} \lambda \left(\text{TE}\right)=\lambda_{\text{N}\text{R}}(\text{TE})+\lambda_{\text{D}\text{S}}\left(\text{TE}\right) \end{array}$$ (9)

**Degree matrix**. According to the results above, the degree matrix **D** was redefined as follows:


$$\begin{array}{c} 𝐃.\text{arcs}[i][j]=\left\{ \begin{array}{c} @l\lambda ({\text{TE}}_{i}),\;\;\;i=j.\\ \text{0},\;\;\text{ otherwise}. \end{array}\right. \end{array}$$ (10)

**Loss function**. Translating target KG into source space can be considered as translating all sub-KGs. For each sub-KG, translated sub-KG was denoted as sub-KG.’ the translated similarity between sub-KG and sub-KG’ can be measured by MTS as Equation 4. Therefore, the loss function *f* of GCN-MTS is maximizing the translate similarities summation of each pair of sub-KG and sub-KG.’ The parameter matrix was denoted as **W**. *f* was defined as follows:


$$\begin{array}{c} \;f(𝐖)=-\sum_{i=\text{1}}^{n}{\text{sim}}_{\text{T}}({\text{sub}-\text{KG}}_{i},\;{\text{sub}-\text{KG}}_{i}\prime ) \end{array}$$ (11)

To sum up, the inputs of GCN-MTS are the pre-trained vectors of TEs by TransE, and the outputs are the new vectors of TEs in source space. The pseudo-codes of overall approach are introduced in Algorithm 1. A new representation of target KG can be obtained by the approach above, who was denoted as KG.’ Finally, alignments were achieved by calculating the distance of each pair of entities between source KG and KG.’

**Algorithm 1. The pseudo-codes of translating target KG into source space.**

 **Input:** Target KG (List<entity> TE), Seed entities (List<entity> SE)

 **Output:** translated KG (List<entity> TTE)

TTE.add(SE) *// translating seed entities*

*// partitioning target KG*

for SE[i] in TTE:

 List<entity> sub[i] = []

end for

for TE[j] in TE and not in TTE:

 for SE[i] in TTE:

  *// Dis(a, b) is the Euclidean distance between a and b*

  if Dis(TE[j], SE[i]) is minimum:

   sub[i].add(TE[j])

  end if

 end for

end for

*// calculating importance coefficient*

for each sub[i]:

 for TE[j] in sub[i]:

  *// λ(a) is the importance coefficient of a*

  calculate λ(TE[j]) by Equation 7 ~ 9

 end for

end for

*// translating target KG*

for k from 0 to n:

 *// W is updated with the loss function as Equation 11*

 TE = *σ*(D^-1/2^·(A + I)·D^-1/2^·TE·W)

end for

TTE.add(each TE[j])

return TTE

## 4. Evaluation

### 4.1. Experimental data and configuration

To evaluate our proposed method, we took **DBpedia** KG (DBP) as source KG, and took **LinkedGeoData** (LGD), **Geonames** (GEO), **YAGO** as target KG. The entity alignment references of DBP-LGD, DBP-GEO, DBP-YAGO had been achieved by DBpedia organization. We selected PERSON, LOCATION, ORGANIZATION entities in KGs above as experimental data, which contain 20000–30000 entities and 100000–200000 relationships respectively. We chose about 30% alignments as seed entities from references randomly. The details of data are introduced in Table 1.

**Table 1:** Description of 3 KG pairs.

Dataset		Entities	Relationships	Seeds
DBP-LGD	DBP	24606	193046	7380
	LGD	25911	175033	
DBP-GEO	DBP	24606	193046	7210
	GEO	22005	148201	
DBP-YAGO	DBP	24606	193046	7420
	YAGO	28378	216639	

we compared our proposed method (GCN-MTS) with MCEA [37], DAGCN [38], and EAMI [39], because they used seed alignments also. We adopted TransE to generate entity vectors as starting data, because the algorithms comparing with our proposed method used TransE also.

### 4.2. Evaluation criteria

We used **Hits@1**, **Hits@10**, and **MeanRank** to evaluate the performances of methods. Hits@*k* (*k* = 1, 10) is the proportion of correctly aligned entities ranked in the top *k* prediction. MeanRank is the mean of the rank of correctly aligned entities. The higher Hits@*k* and lower MeanRank indicates the better performance of method.

### 4.3. Experimental hyperparameters

We took 60% of experimental data as train dataset, 20% as validation dataset and 20% as test dataset. All seed alignments are exclusively from the training data. We used grid search to find the best hyperparameters for GCN-MTS on the validation dataset. We chose the learning rate of the Adam optimizer among {0.01; 0.001; 0.0001}, and the *η*_1_ in Equation 2 between [0.01, 0.99] with a step size of 0.01. The *η*_2_ in Equation 2 was determined as 1-*η*_1_. We trained the GCN-MTS with a batch size of 128 and a maximum of 500 epochs. As shown in Fig 7, across all 6 datasets, the optimal value of learning rate remained consistent at 0.001, and the optimal values of *η*_1_ were 0.63, 0.63, 0.63, 0.68, 0.61, and 0.69, respectively. Accordingly, we adopted their average value 0.65 as the final optimal value of *η*_1_., and the final optimal value of *η*_2_ was 0.35.

**Fig 7:**
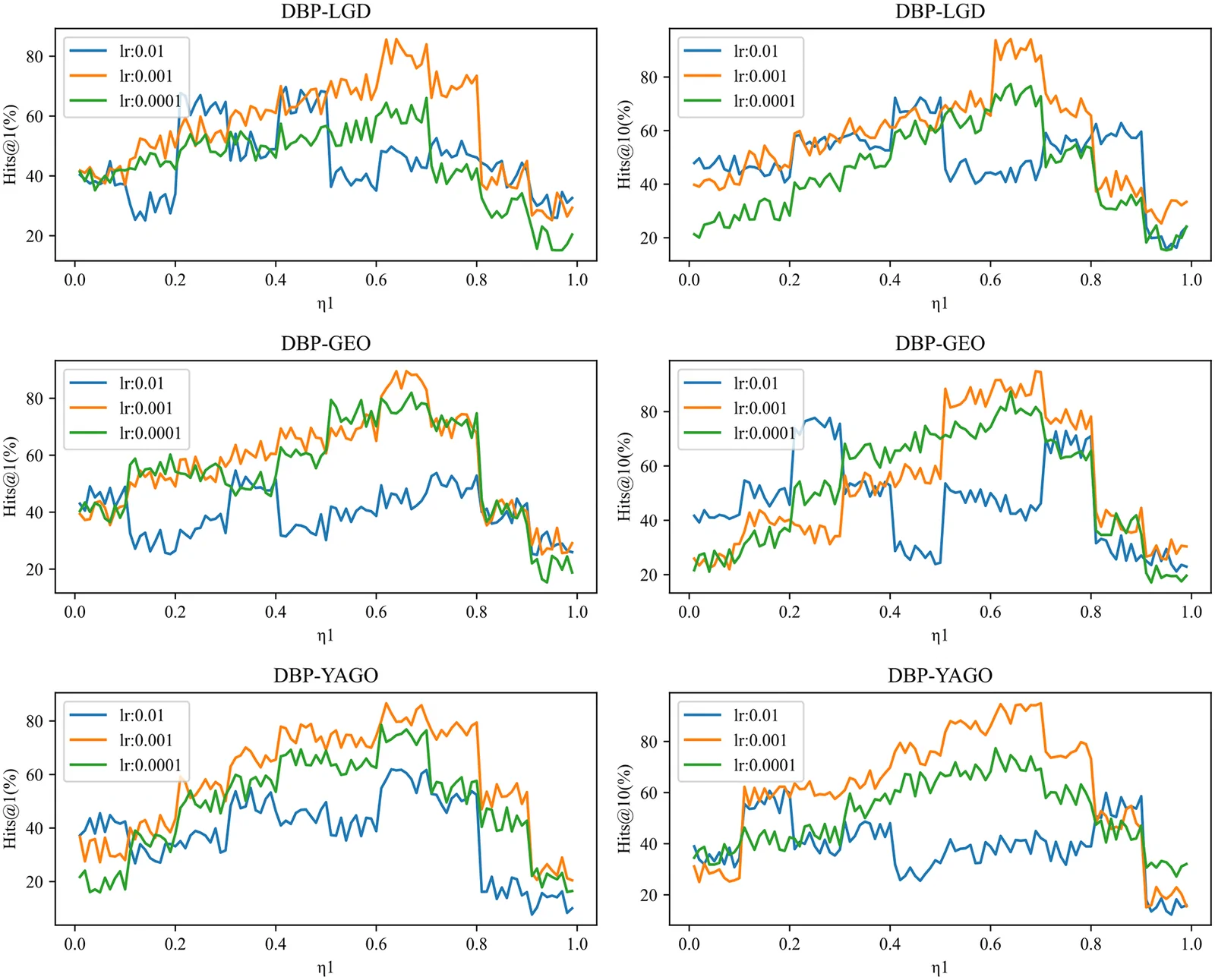
Optimal value of hyperparameters on validation dataset.

When GCN-MTS was configured with the optimized hyperparameters, the loss function tends to converge after 400–450 epochs, as shown in Fig 8, which demonstrated excellent stability and generalization robustness.

**Fig 8:**
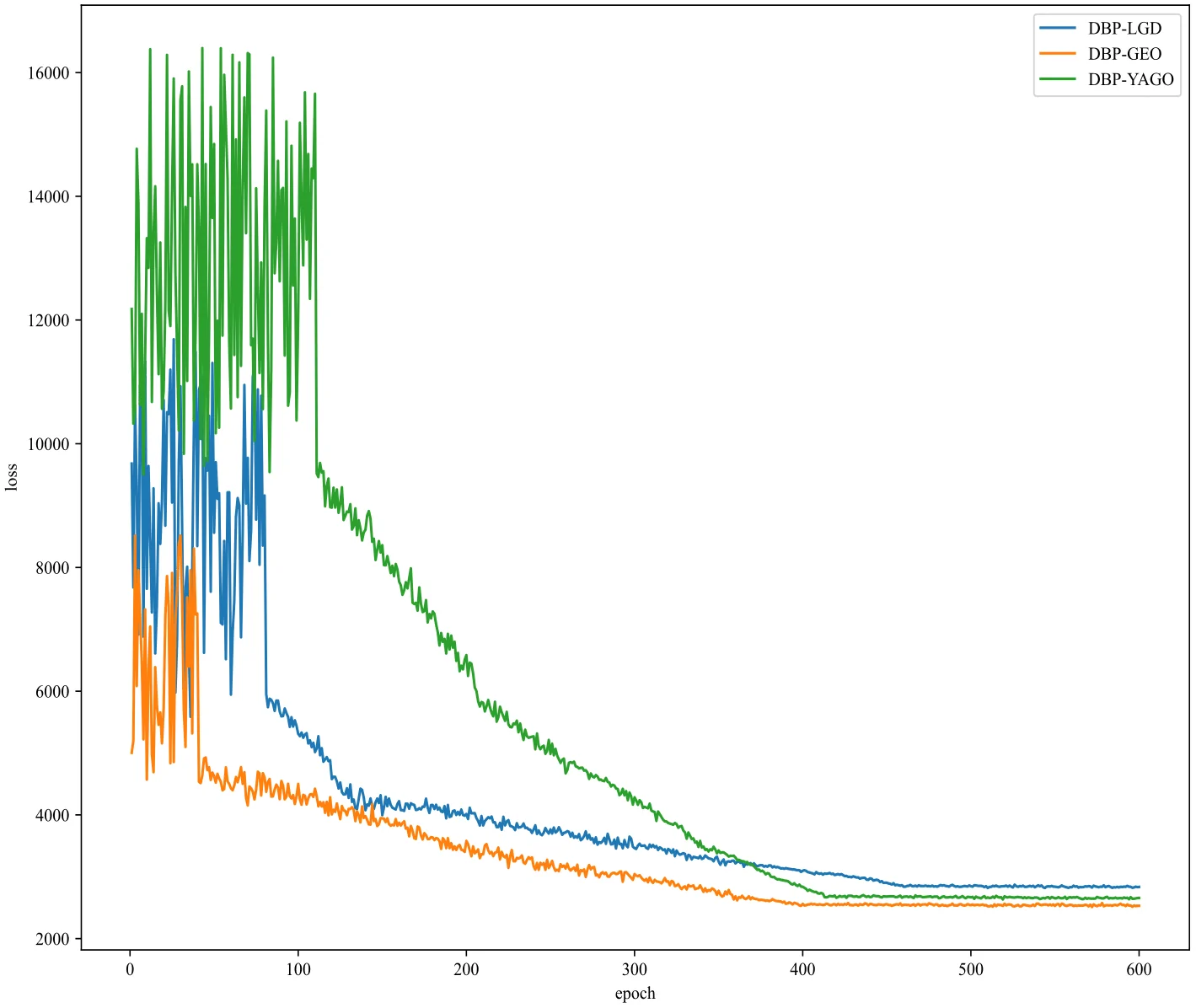
Loss variation over training epochs.

### 4.4. Evaluation results

We executed each program 10 times in same condition, and then the average results were compared with each other. As shown in Fig 9 and Table 2, the results of GCN-MTS were the best, except the Hits@1 of DBP-LGD and the Hits@10 of DBP-YAGO. It was verified that our method GCN-MTS is efficient and has better performance.

**Table 2:** Results of Hits@1, Hits@10, and MeanRank of 4 methods on 3 KG pairs.

Method	DBP-LGD			DBP-GEO			DBP-YAGO		
	Hits@1	Hits@10	MeanRank	Hits@1	Hits@10	MeanRank	Hits@1	Hits@10	MeanRank
MCEA	56.16	63.77	354	66.53	71.46	520	74.78	83.16	126
	66.49	74.45	324	63.42	68.15	538	73.30	76.78	135
	56.80	68.92	340	65.68	69.26	536	75.94	80.35	128
	60.67	71.52	336	63.82	74.60	515	73.15	75.56	144
	59.89	62.53	356	62.36	67.29	561	77.54	83.36	120
	61.58	71.87	327	69.09	72.17	517	81.57	83.55	112
	60.92	76.41	322	71.51	75.47	513	74.99	77.01	130
	65.03	68.55	342	62.43	69.75	527	76.22	83.31	125
	65.32	71.14	338	64.08	70.89	525	69.65	82.12	128
	64.23	66.55	345	64.59	69.66	532	71.45	89.35	109
Average	61.71	69.57	338	65.35	70.87	528	74.86	81.46	126
DAGCN	76.56	77.03	219	70.67	76.62	255	73.84	91.34	121
	72.74	79.07	212	68.04	70.80	281	75.53	94.92	111
	75.42	84.50	198	67.75	75.41	265	73.92	88.85	146
	74.70	82.97	203	68.40	71.73	279	77.70	89.66	139
	72.91	83.83	202	72.01	75.37	266	77.69	90.70	127
	77.01	84.30	201	71.07	75.41	263	80.13	88.61	153
	70.15	73.47	220	70.22	74.37	273	74.44	89.70	137
	73.74	79.07	209	69.43	76.28	261	79.51	94.64	112
	71.24	80.76	207	73.84	78.10	240	78.32	92.52	119
	73.23	78.37	214	71.13	73.80	274	75.39	91.57	121
Average	73.77	80.34	209	70.26	74.79	266	76.65	**91.25**	129
EAMI	84.12	87.08	135	75.24	77.36	204	77.28	88.40	139
	81.04	83.17	146	73.28	83.57	190	70.99	86.20	149
	82.53	86.31	139	74.95	76.92	206	72.92	86.71	143
	80.35	86.16	140	75.34	81.60	194	81.04	89.03	131
	81.29	89.96	131	79.54	81.57	196	76.69	88.75	132
	81.80	82.33	149	77.95	86.04	183	72.84	88.29	139
	80.24	82.47	148	76.70	81.86	192	79.43	81.35	150
	82.71	86.97	135	74.87	84.64	184	75.25	87.56	143
	76.66	88.43	133	76.10	89.82	169	77.63	91.03	116
	81.23	83.80	141	77.34	79.89	202	75.31	87.91	141
Average	**81.20**	85.67	140	76.13	82.33	192	75.94	87.52	138
GCN-MTS	81.49	88.20	69	85.29	93.68	42	81.51	91.84	74
	84.13	87.34	73	85.15	89.95	52	84.10	92.40	66
	84.79	90.03	64	81.39	87.41	63	83.12	90.82	80
	80.63	91.14	63	87.42	91.46	50	84.59	89.17	102
	79.85	88.64	67	91.20	93.48	47	81.01	91.20	76
	83.61	92.65	61	85.61	88.74	55	81.20	89.27	98
	80.80	89.75	67	83.68	88.43	56	85.45	89.54	90
	76.40	86.19	76	83.74	92.34	49	85.53	91.55	75
	78.60	92.14	62	85.08	88.35	60	82.66	89.96	88
	76.08	87.34	74	85.88	86.31	66	80.19	90.32	87
Average	80.64	**89.34**	**68**	**85.44**	**90.02**	**54**	**82.94**	90.61	**84**

**Fig 9:**
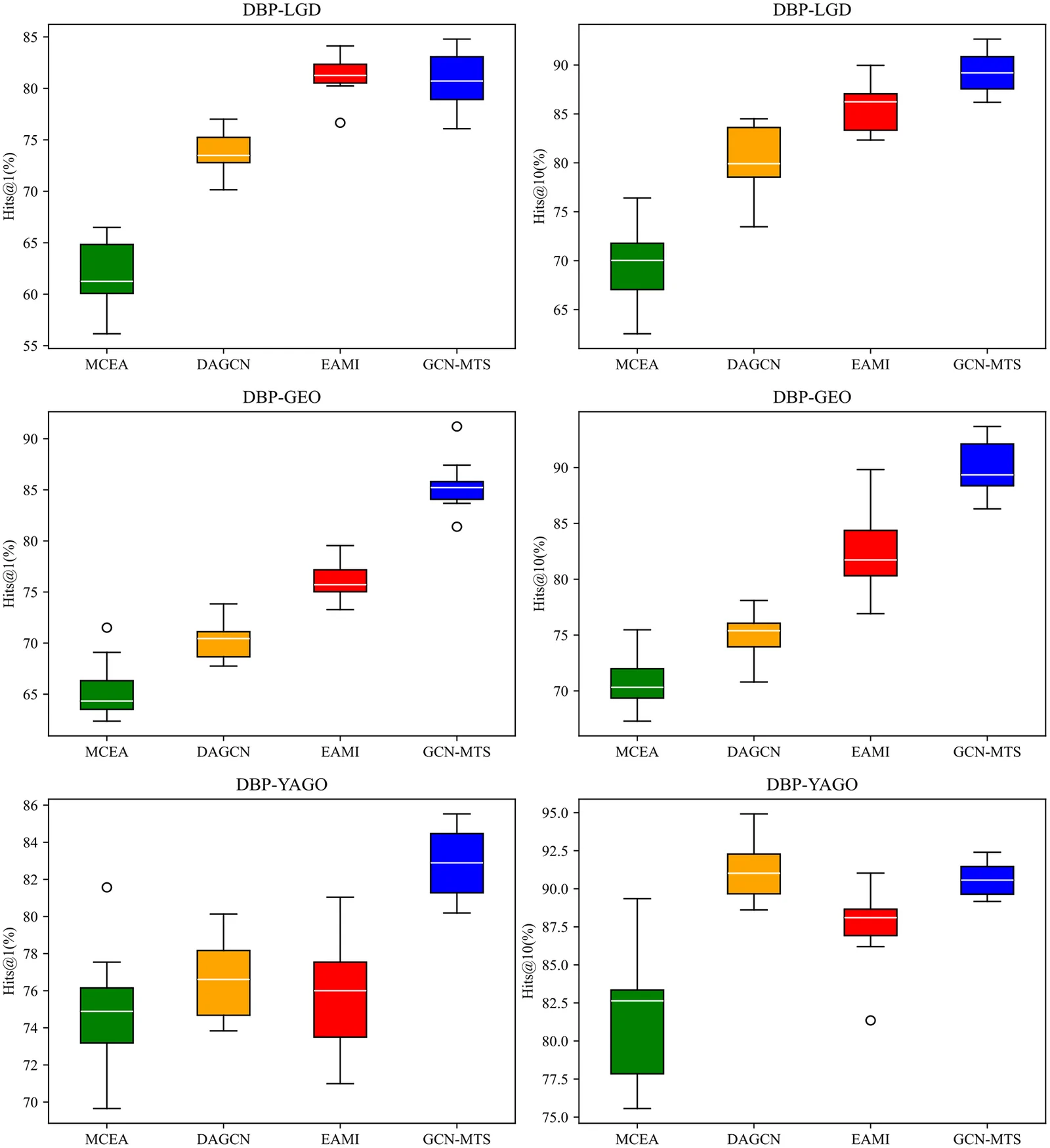
Box plots of Hits@1 and Hits@10 of 4 methods on 3 KG pairs.

To verify the significance of differences in experimental results, we conducted an analysis of variance (ANOVA) on the experimental data, with different 4 methods defined as factors. ANOVA was performed separately on Hits@1, Hits@10, and MeanRank among 4 methods. The null hypothesis for all tests assumed equal population means. As shown in Table 3 and Table 4, the results revealed that the p-values of each test were far less than 0.05. Therefore, ANOVA indicated statistically significant differences among the experimental results.

**Table 3:** Descriptive Statistical Analysis results on Hits@1, Hits@10, and MeanRank among 4 methods on 3 KG pairs.

Grouping metric	Methods	Observations	Mean	Variance	95% CI		Max	Min
					Lower	Upper		
Hits@1 on DBP-LGD	MCEA	10	61.71	12.58	59.17	64.25	66.49	56.16
	DAGCN	10	73.77	4.83	72.20	75.34	77.01	70.15
	EAMI	10	81.20	3.91	79.78	82.61	84.12	76.66
	GCN-MTS	10	80.64	9.17	78.47	82.80	84.79	76.08
Hits@10 on DBP-LGD	MCEA	10	69.57	19.55	66.41	72.73	76.41	62.53
	DAGCN	10	80.34	13.06	77.75	82.92	84.50	73.47
	EAMI	10	85.67	6.83	83.80	87.54	89.96	82.33
	GCN-MTS	10	89.34	4.71	87.79	90.89	92.65	86.19
MeanRank on DBP-LGD	MCEA	10	338.40	136.04	330.06	346.74	356.00	322.00
	DAGCN	10	208.50	58.50	203.03	213.97	220.00	198.00
	EAMI	10	139.70	40.23	135.16	144.24	149.00	131.00
	GCN-MTS	10	67.60	28.04	63.81	71.39	76.00	61.00
Hits@1 on DBP-GEO	MCEA	10	65.35	8.80	63.23	67.47	71.51	62.36
	DAGCN	10	70.26	3.65	68.89	71.62	73.84	67.75
	EAMI	10	76.13	3.25	74.84	77.42	79.54	73.28
	GCN-MTS	10	85.44	6.66	83.60	87.29	91.20	81.39
Hits@10 on DBP-GEO	MCEA	10	70.87	6.96	68.98	72.76	75.47	67.29
	DAGCN	10	74.79	4.90	73.21	76.37	78.10	70.80
	EAMI	10	82.33	15.33	79.53	85.13	89.82	76.92
	GCN-MTS	10	90.02	6.71	88.16	91.87	93.68	86.31
MeanRank on DBP-GEO	MCEA	10	528.40	206.27	518.13	538.67	561.00	513.00
	DAGCN	10	265.70	148.68	256.98	274.42	281.00	240.00
	EAMI	10	192.00	126.44	183.96	200.04	206.00	169.00
	GCN-MTS	10	54.00	56.00	48.65	59.35	66.00	42.00
Hits@1 on DBP-YAGO	MCEA	10	74.86	11.04	72.48	77.24	81.57	69.65
	DAGCN	10	76.65	5.37	74.99	78.30	80.13	73.84
	EAMI	10	75.94	9.72	73.71	78.17	81.04	70.99
	GCN-MTS	10	82.94	3.73	81.55	84.32	85.53	80.19
Hits@10 on DBP-YAGO	MCEA	10	81.46	17.19	78.49	84.42	89.35	75.56
	DAGCN	10	91.25	4.96	89.66	92.84	94.92	88.61
	EAMI	10	87.52	6.46	85.71	89.34	91.03	81.35
	GCN-MTS	10	90.61	1.28	89.80	91.42	92.40	89.17
MeanRank on DBP-YAGO	MCEA	10	125.70	105.57	118.35	133.05	144.00	109.00
	DAGCN	10	128.60	208.04	118.28	138.92	153.00	111.00
	EAMI	10	138.30	99.34	131.17	145.43	150.00	116.00
	GCN-MTS	10	83.60	129.38	75.46	91.74	102.00	66.00

**Table 4:** ANOVA results on Hits@1, Hits@10, and MeanRank among 4 methods on 3 KG pairs.

Grouping metric	Source	SS	df	MS	F	P-value	F crit
Hits@1 on DBP-LGD	SSA	2465.50	3	821.83	107.8126	**4.78E-18**	2.87
	SSE	274.42	36	7.62			
	SST	2739.92	39				
Hits@10 on DBP-LGD	SSA	2222.30	3	740.77	67.11	**8.17E-15**	2.87
	SSE	397.35	36	11.04			
	SST	2619.65	39				
MeanRank on DBP-LGD	SSA	398682.50	3	132894.17	2022.57	**3.64E-40**	2.87
	SSE	2365.40	36	65.71			
	SST	401047.90	39				
Hits@1 on DBP-GEO	SSA	2239.80	3	746.60	133.59	**1.45E-19**	2.87
	SSE	201.19	36	5.59			
	SST	2440.99	39				
Hits@10 on DBP-GEO	SSA	2152.28	3	717.43	84.66	**2.25E-16**	2.87
	SSE	305.06	36	8.47			
	SST	2457.33	39				
MeanRank on DBP-GEO	SSA	1191310.48	3	397103.49	2955.80	**4.07E-43**	2.87
	SSE	4836.50	36	134.35			
	SST	1196146.98	39				
Hits@1 on DBP-YAGO	SSA	396.56	3	132.19	17.71	**3.15E-07**	2.87
	SSE	268.77	36	7.47			
	SST	665.33	39				
Hits@10 on DBP-YAGO	SSA	600.91	3	200.30	26.81	**2.74E-09**	2.87
	SSE	268.99	36	7.47			
	SST	869.90	39				
MeanRank on DBP-YAGO	SSA	17626.90	3	5875.63	43.34	**4.9E-12**	2.87
	SSE	4881.00	36	135.58			
	SST	22507.90	39				

### 4.5. Ablation study

We conduct ablation studies to verify the effectiveness of the core proposed module MTS. 30% pairs of entity as seed alignments were selected randomly. TransE, TransH, TransR were deployed to generate the original entity embedding, GCN and GCN-MTS were deployed to enhance the embeddings in same vector space, respectively. As shown in Fig 10, the average Hits@1 of TransE, TransH, and TransR on 3 KG pairs were increased by 25.95, 27.81, and 20.50, respectively. The average Hits@10 of TransE, TransH, and TransR on 3 KG pairs were increased by 22.22, 16.48, and 20.92, respectively. Comprehensively, MTS improves approximately 22 percentage points on GCN.

**Fig 10:**
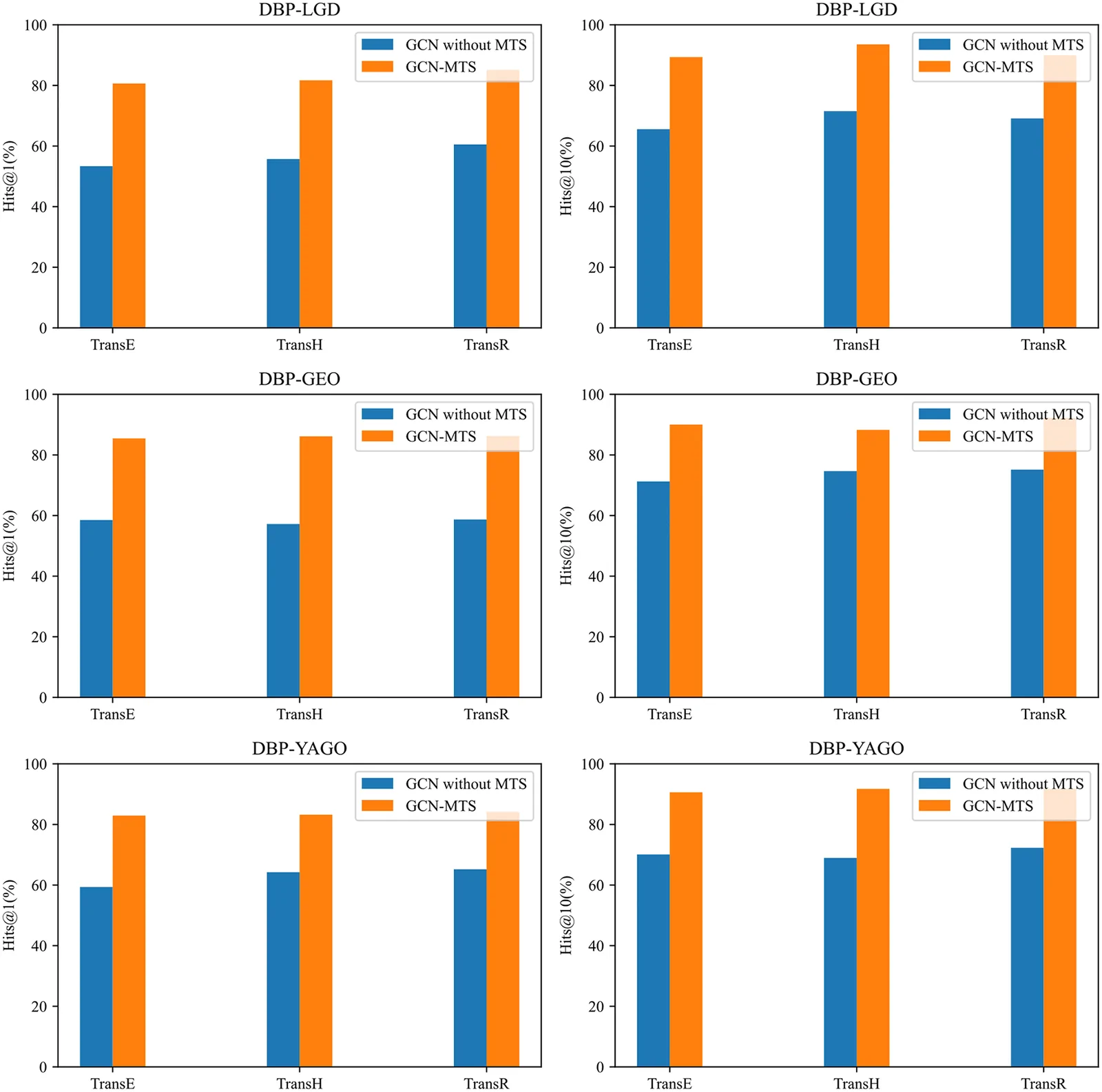
Comparison results between GCN without MTS and GCN-MTS (TransE, TransH, TransR).

To demonstrate the performance of GCN-MTS more comprehensively, we took other experiments by changing seed alignments.

We still selected 30% pairs of entity as seed alignments, but different seed entities were selected, which had different numbers of skip between seed entities in target KG. We took MeanSkip as the criteria of the numbers of skip, that was the mean of skips between any two entities. As shown in Fig 11, the lower numbers of skip led to the worse performance of method. However, GCN-MTS kept in its better performance, i.e., GCN-MTS has better consistency while the seed alignments were changed. Especially with lower MeanSkip, GCN-MTS shows better performance significantly.

**Fig 11:**
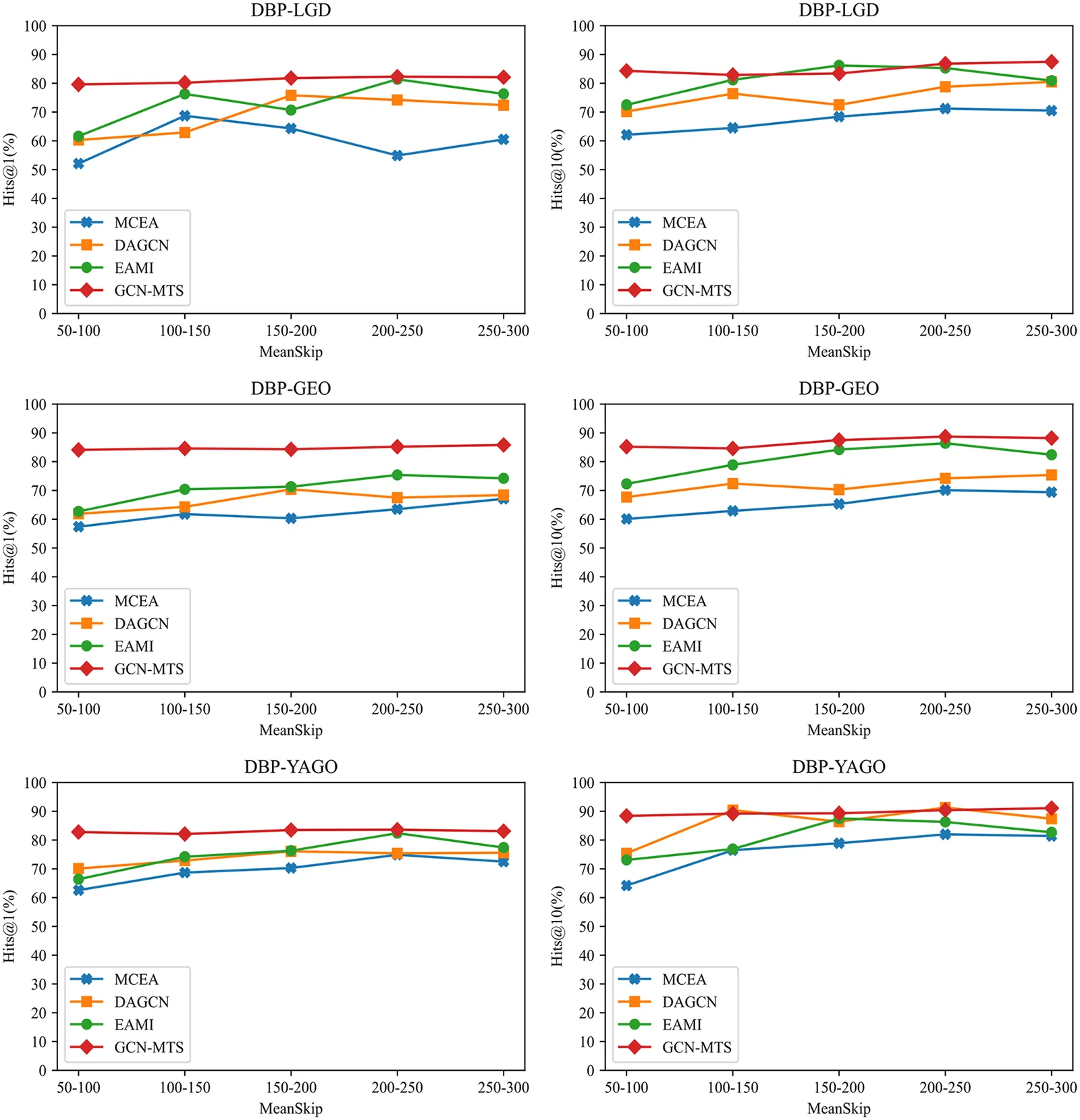
Results of 4 method by changing numbers of skip between Seeds (50 ~ 300).

We tested the performances of each method with different numbers of seed alignments, that was 10%−50%. We executed each program 10 times, and then took the average results. As shown in Fig 12, the higher proportion of seed alignments led to the higher performance of method. However, GCN-MTS showed much better than other methods with fewer seed alignments, such as 10% and 20%.

**Fig 12:**
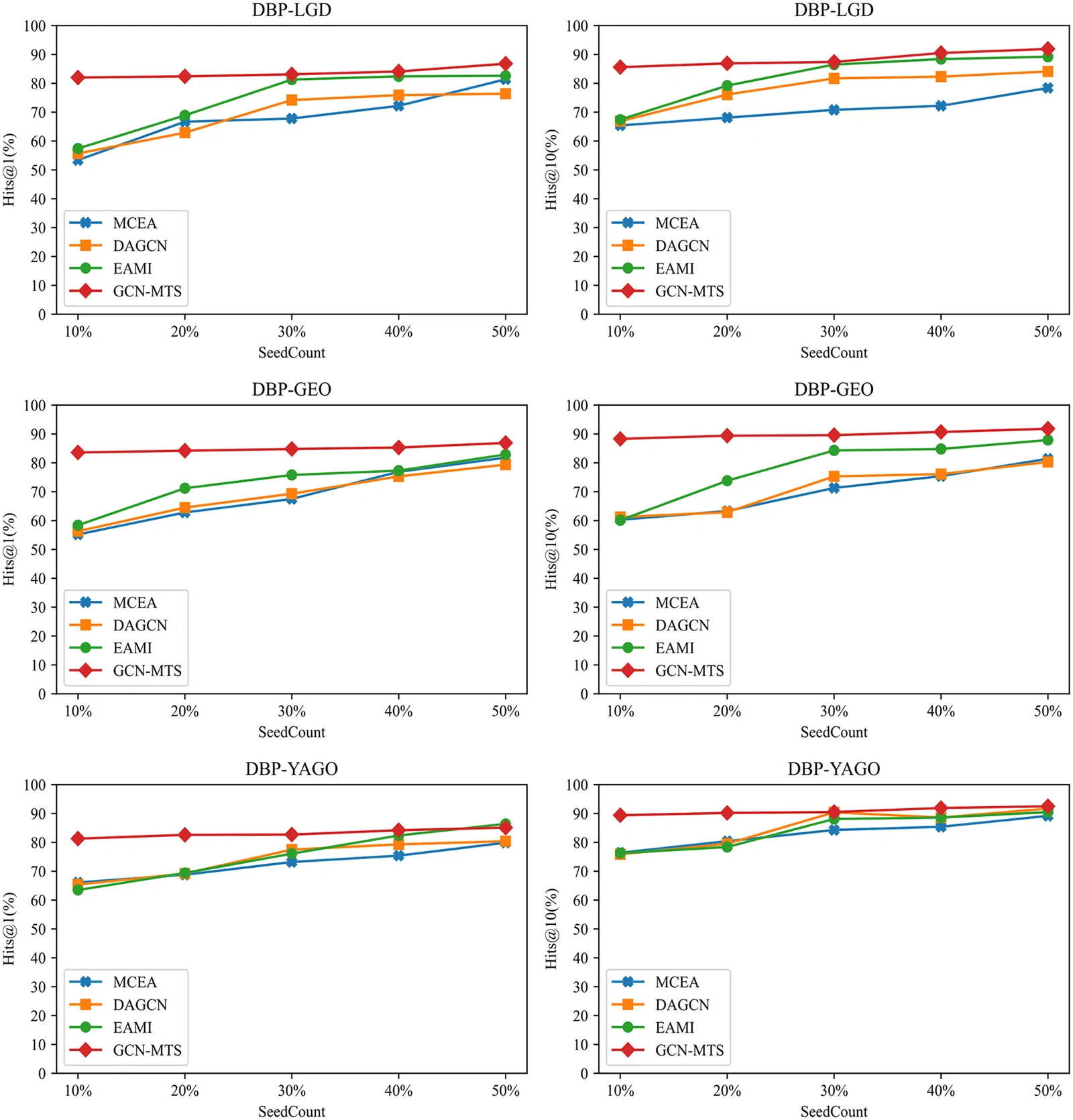
Results of 4 method by changing numbers of Seeds (10% ~ 50%).

## 5. Discussion

In the previous research, entity alignment was achieved by various deep learning models mostly. The performances of these models were good, especially of GCN model. Because of that, we selected GCN as the basic model in this paper. However, we studied the previous GCN models, and tested the alignment result. The alignments that had not been recalled successfully had been surveyed as the emphases. We found that some entity embeddings had been changed too much when they are embedded into the space of the other KG. For example, in target KG T, entity A and entity B were correlated strongly. The distance between the embedding of A and the embedding of B was short also. But when T was embedded into the space of the source KG, the distance between A and B was no longer short. In this situation, there was a high probability that A or B could not been recalled.

If many entity embeddings were changed too much, i.e., the structure of KG was changed too much, the recall of entity alignment was descended obviously. Inspired by this, we attempted to preserve the structure of target KG when it was been embedded into the space of source KG. Under the influence of Seeds in source KG, preserving all the entity embeddings was impractical. So, we decided to preserve the most entity embeddings not been changed too much. To achieve this, we partitioned target KG into several sub-KGs. Each sub-KG had only one Seed, the other entities in sub-KG were affected by only one Seed, so the change of them was no longer too much. The structure of each sub-KG was preserved, and the performances of entity alignment was enhanced. This work was achieved by GCN-MTS.

However, the edge entities of each sub-KG still faced the same problem mentioned above. How to preserve the edge entities is the next research direction as our works.

## 6. Conclusions

To improve performance of embedding based entity alignment, this paper proposed a novel method named GCN-MTS. While GCN-MTS translated target KG into the vector space of source KG, the relative embeddings structure of relationships in target KG was maintained as far as possible by GCN-MTS. The adjacent matrix, degree matrix and loss function of GCN-MTS were enhanced by MTS. Comparing with three the state of art methods and taking three KG pairs as experimental data, three groups of experiments were conducted to evaluate GCN-MTS. The results showed that GCN-MTS had higher performances including Hits@1, Hits@10 and MeanRank. Meanwhile, GCN-MTS was more stable in the condition of changing seed alignments or changing numbers of seed alignments.
